# Extra Supplement.—The Nursing Mirror

**Published:** 1893-12-16

**Authors:** 


"The Hospital" Nursing Mirror.

Being the Extra Nursing Supplement of "The Hospital" Newspaper.

[Contributions for this Supplement should be addressed to the Editor, The Hospital, 428, Strand, London, W.C., and should have the word "Nursing" plainly written in left-hand top corner of the envelope.]

### News from the Nursing World.

### THE NOVEL COMPETITION FOR NURSES.

Intending competitors are reminded that plans and descriptions of the Model Home for Nurses must reach Dr. Billings, Surgeon-General's Department, Washington, D.C., U.S.A., not later than January 1st, 1894.

### CHRISTMAS FESTIVITIES.

We beg to remind our readers that due notice of festivities and descriptions intended for insertion in The Hospital must reach the office in good time. We are delighted to spare space in our columns for chronicling as many as possible of the entertainments given to patients and nurses, but naturally they are only suitable for the Christmas season, and we cannot permit them to exceed certain definite limits. It will therefore be of mutual advantage if all members of our large circle of correspondents will aid us by sending in their contributions immediately after each event. Any approaching festivities of which preliminary announcements are desired must be officially reported to us ten days at least before they are to take place.

### NIGHT AND DAY.

There are many points on which nurses and guardians hold different views, and one of the most important is night duty. It is quite exceptional for the nurse to be called up at night, asserted a gentleman the other day at a Board meeting. It did not apparently occur to the speaker that consideration to the nurse's rest could only be shown at the expense of the patients' safety. If the only trained nurse in a workhouse be also the only midwife, she must be frequently disturbed at night, and yet has to do responsible work all day. If a trained nurse is needed for the twelve hours of daylight, how much more is she required during the long night watches? If every guardian, who goes to his own comfortable bed with the reflection that the weary workhouse nurse is not often disturbed, would just think instead how many babies are born, and what numbers of sick people die every night, conscience might well banish sleep.

### POISONS.

Accustomed to see poisons kept under lock and key in a hospital, nurses are often dismayed at the casual manner in which they are left about in private houses. The district nurse, too, is much exercised in her mind by the frequency with which she finds lotions and liniments kept in close proximity to the patient. Some of the recent suggestions as to roughened or coloured bottles and conspicuous "poison" labels appear well worthy consideration. The proposal that "carbolic acid should be added to the schedule of poisons within the meaning of the Pharmacy Act" cannot be too highly commended. In any case, it is the immediate duty of all nurses to show by example and precept what extreme care should invariably be observed with regard to the custody of dangerous drugs.

### OFF TO ST. HELENA.

Miss Blennerhassett sends us a farewell greeting off Finisterre, from the U.S.S. "Gaul." She has unfortunately been very ill of late with fever and ague, which has prevented her from doing many things she wished to carry through before sailing. Miss Nightingale has been greatly interested in the work of the two nurses in Mashonaland, and wrote them a delightful letter of sympathy and approval just before they left, which was accompanied by an offering of flowers and laurel leaves. Mashonaland has produced two heroines, whose conduct and character make them prominent amongst the most worthy representatives which modern nursing has yet given to the world. Without advertisement, with becoming modesty, with an intrepidity of spirit and courage as rare as it is admirable, Miss Blennerhassett and Miss Sleeman are proud to be nurses, and nurses may well be proud both of them and their work. An eminent authority on nursing, having made an appointment with these ladies, told her maid she expected them. The maid said she always understood that they went about with two lions, and would it be safe to shut them up in the dining-room? This story points a moral, which it would be well if some of the self-advertisers, who at present affect nursing, would take to heart. Had the maid been told to expect any two members of this latter set, she would not have said she heard they always went about *with* two lions, but *as* two lions—a very different matter. We wish our friends the best of times in St. Helena, and we hope to welcome them back in perfect health and the best of spirits in due course.

### NURSERIES FOR THE R.B.N.A.

We wonder if the governors of the Sussex County Hospital, Brighton, are aware that the funds of their institution in future may be applied in part to the production of members of the Royal British Nurses' Association? Any probationer who enters at the Sussex County Hospital is apparently to be carefully nurtured until the end of the third year, when she is to receive £5 premium if she enrolls herself on the register of the R.B.N.A. The English are, or were, a high-spirited and independent nation. We wonder if the younger generation of women will consent to be baited under the Brighton system? We cannot believe that the Council of the R.B.N.A. can be any party to the new rules at the Sussex County Hospital, as they must tend to make the Association unpopular with nurses, whilst they will excite keen hostility amongst the responsible managers of hospitals all over the country. Religious intolerance would be a mere trifle compared with the intolerance of matrons who, being members of the R.B.N.A., are charged with a mission on behalf of the Association. Whatever course the Association may feel called upon to take, in the present instance, we believe the general public, as represented by the governors and committees of our hospitals, will regard any such attempt to interfere with the liberty of individual members of the nursing staff on the Brighton plan as evidence that they must be most careful, when selecting their matrons, to secure ladies who are quite independent of any such outside control.

### "THE USUAL EXTRAS."

Are baths actual necessaries or mere luxuries? may well be asked by those who find them set down as "extras" to be paid for each time of using. Nurses accustomed to be made free of the commodious bathrooms in their training school homes find that in private homes for nurses the system is altogether different. The price seems to vary from twopence to sixpence per bath, but it would surely be more satisfactory to dispense with this tax and certainly to include water (and plenty of it) in the regular charges. Only superfluities can fairly be set down as "extras," except by lodging-house keepers at the sea, who say truly, "We've got to make what we can out of the visitors during our short season."

### ISOLATION IN A TENT.

The experiment of isolating small-pox patients in a tent is being tried at Leicester. To keep up uniform temperature appears the chief difficulty so far. Infectious hospitals, of whatever materials composed, generally seem to err on the side of over, not under, ventilation, and the long open corridors are sufficiently dreaded by staff and convalescents. Perhaps some day a happier medium will be arrived at between the air-tight chambers of the old and draughty dwellings of the modern day. Even a tent might compare favourably with some kinds of erections.

### TARDY REFORM.

The inmates of Newton Abbot Infirmary owe a debt of gratitude to Dr. Ley for his brave advocacy of their claims to proper care and attention. Sad abuses have been hushed up there for a long time past, but public opinion is now "concerning itself with the matter." A motion to advertise for a second nurse was carried at the last Board meeting in spite of the suggestion by one of the guardians that a nursing staff could be formed out of the able-bodied paupers. Most of these so-alled "able-bodied" persons are ill, and we hear that one of them at any rate "appeared daft"—sitting beside a bed whose proper occupant, a recently-confined woman of weak intellect, was discovered by the doctor wandering about the corridor in her night-dress. This is but one of the incidents recently brought to light, and the suffocation of a baby and death of a woman are others about which at the time they occurred the Board "closed their mouths so that the reporters should not hear of it."

### A NURSES' HOME.

A home for the Belfast district nurses is likely to be an accomplished fact ere long, for one of the vice-presidents has given £500 towards the £1,000 required for its erection. The work was first started by Miss Macpherson, and the subscription list now opened bears the name of the Macpherson Fund.

### AN ILLOGICAL DEDUCTION.

Nearly a hundred applications were recently made in Sydney for the matronship of the Thomas Walker Convalescent Home, and this fact has served as a text for many homilies on the crowded state of the nursing profession. Yet the two things have but little bearing on each other. Even in England, where trained nursing has made enormous progress, not only are there still untrained matrons at the head of some of the nursing institutions, but amongst the candidates for every vacant post there are always persons whose suitability for work begins and ends with the obligation to earn their own living. An advertisement of fairly remunerated work always receives crowds of answers which prove the willingness but not the ability of the applicants. Therefore it is hardly likely that more than a small proportion of the would-be matrons at Sydney were in any way qualified persons. Many under-nursed workhouse infirmaries as well as the fever hospitals daily show sadly enough that there is little fear of nursing being an over-stocked profession, in England at any rate, for many a day. All trained women who have proved themselves competent nurses find a choice of useful careers open before them.

### BALTIMORE.

The Nurses' Journal Club at Johns Hopkins Hospital has developed after a most satisfactory fashion, great interest and spirit being evinced in the discussions which follow the reading of the papers. The meetings are held twice in each month, the articles given being sometimes original, whilst on other occasions they are those which have previously appeared in American and English journals. The success of this club should encourage other hospitals to organise similar ones, for discussions are obviously advantageous to nurses, who thus become acquainted with the progress of their profession in their own and in other countries.

### NOVEL METHODS.

The pathetic attempts made by the poorest class of hospital patients "to be kept in" a little longer are as well known as the wonderful variety of ailments which they invariably discover themselves to be possessed of when the day of departure draws near. But apparently quite strong measures would find favour sometimes in India. In the Victoria Hospital at Benares, for example, a patient on leaving actually asked for medicine to make her ill, as she wanted to return there in a few days, and she managed, without the aid of the coveted physic, to get herself rapidly into a condition which really necessitated her being readmitted. If such proceedings are hardly commendable, the appreciation shown of the comforts of hospital life is certainly undeniable.

### SHORT ITEMS.

The question of better accommodation for the nurses at Islington Infirmary has been referred to the House Committee.—Three Lincolnshire County Councils have decided to offer sick nursing scholarships in connection with the Rural Nursing Association.—Lady Jeune opened a Home at Nutsford Place, Edgware Road, on the 8th inst., in connection with the Church Army. The house will accommodate eighteen mission nurses.—A conversazione, in aid of the funds of the Bramley and District Nursing Society was recently held at Bramley Town Hall, and was well attended. The Society is affiliated with the Queen's Jubilee Institute.—Professor Dewar will give six lectures at the Royal Institution during the holidays, adapted to a juvenile audience, on the subject of Gaseous and Liquid Air.—Seventy-five thousand pounds has been spent on the Walker Convalescent Hospital at Sydney, which will accommodate about sixty persons.—Dr. Gourley has given gratuitous ambulance instruction to about 500 men at Hartlepool.—Some well-attended nursing lectures have been given at Cheshunt, Herts, by Miss Cullip, Sister-in-charge of the Cottage Hospital. Miss Cullip was trained at the London Hospital, and is an excellent nurse.

### On the Nursing of Diseases of the Stomach.

VI.—DYSPEPSIA (*continued*).

The symptoms of dyspepsia may be divided into those which may be referred to the stomach, and those which may be referred to other organs.

The symptoms which accompany dyspepsia are to a large extent the same as those which occur in the various diseases of the stomach. In dyspeptic patients the appetite is very variable. In some cases it is little affected, in others there is loss of appetite. Frequently there is a craving for food, which is satisfied after taking a very small quantity. There may, or may not be, much thirst, according to the kind of dyspepsia. There is generally pain and uneasiness. Pain may be referred to the pit of the stomach or some neighbouring part, as between the shoulders. Often the pain is described as going through from front to back. In many cases the pain is increased by taking food either directly, or after some hours. There may be a feeling of fulness, or shooting, burning, or aching pain. Flatulence and eructation are as a rule present. The amount of "wind" discharged may be very great. Nausea and vomiting are common symptoms. With regard to other organs, the tongue is often furred, cracked, or fissured, or may be red with prominent papillæ, In the common form of dyspepsia, the bowels are constipated, but in a rarer form there may be diarrhœa. There is often palpitation or irregular action of the heart, with some difficulty in breathing. There may be affections of the skin, as nettle-rash, or redness of the nose and cheeks, with pimples. There is very frequently headache, lassitude, and depression of spirits; and, in the more severe forms, weakness and loss of flesh.

The treatment of dyspepsia is often of considerable difficulty. Of course, the first thing is to determine as far as possible what is the cause of the dyspepsia. Attention to diet is certainly by far the most important part of the treatment It is necessary to regulate the amount of food taken, and its kind, not forgetting the proper intervals at which food is taken, and the conditions under which it is eaten. Also, it is necessary to see that the teeth are in good order, so that the food can be properly masticated. In some few cases a little alcohol taken with food may aid digestion, but in very many cases it is essential that no alcohol be taken. And this is the rule not only in those cases in which alcohol is the primary cause of the indigestion, but in other cases as well. Loss of appetite may be a symptom requiring treatment. In some cases bitter tonics are useful, as gentian, calumba, with sodium bicarbonate, or tonics, such as quinine, strychnine, iron. If constipation is present it must be treated.

Pain accompanying dyspepsia requires treatment varying according to the circumstances under which it arises. In cases of pain occurring after food sedatives are often useful, as bismuth and hydrocyanic acid. If the pain is dependent on distension, with flatulency; peppermint, ginger, or other substances of similar nature are ordered. Opium is required in some cases, also blisters or fomentations to the epigastrium may give relief. The treatment of nausea or vomiting has already been considered. It is to be mentioned also that in cases of chronic dyspepsia all hygienic measures are important, as suitable exercise, well ventilated rooms, regular hours, change of air.

This is not the place to discuss the treatment either of cases due to organic disease of the stomach, or of the more severe and intractible forms of indigestion.

### Wants and Workers.

The Matron of the West Norfolk and Lynn Hospital proposes, with the help of her friends, giving a tea to 330 out-patients on December 28th, and on the following Satuıday a Christmas tree and concert will be given to the hospital in-patients. Any gifts of clothing, toys, or money will be most gratefully acknowledged by the Matron, West Norfolk and Lynn Hospital, King's Lynn.

### Nursing in Labrador.

(By Our Own Correspondent.)

THE END OF THE SEASON.

When the time drew near for us to leave Battle Harbour the leave-takings were really most touching. Women wept, and men wrung our hands, turning their heads away that we might not be saddened by the grief they exhibited. "We shall be so terribly lonesome," they said, "we shall count every day till you come back in the spring." Two thousand two hundred patients have been treated by the three doctors during their various tours, and many of these were serious cases. No doctors hitherto being available, the poor folks have forlornly "got used to" being without medical aid, and their appreciation of the help recently brought to them by means of the D.S.M.F. is correspondingly great. It is to be hoped that the present admirable scheme will enlarge annually, so that each season will in future be marked by more workers and greater stores of medical and other comforts being sent out to the brave men and their families on these desolate fishing stations.

Qualified doctors and good nurses certainly find a noble field of labour awaits them on the Labrador, and their ministrations are as valued as they are valuable. When a Newfoundlander dies there it is the custom to place him a coffin in which a layer of salt has been spread. Then the body, with the exception of the face, is entirely covered with salt, and at the end of the season it is taken back to Newfoundland and buried with considerable ceremony. Only natives are interred on the Labrador in a cemetery situated in a deep ravine surrounded by three very high rocks and by the open sea. And very wonderful the sea is here; it is difficult to describe its grandeur—waves dashing over the rocks in leaps and bounds, and sending up spray forty or fifty feet above them. It seems impossible to believe that very soon this seething ocean will be still, frozen so hard that people will walk on it.

The "Albert" had met with very rough weather before she rejoined us, having once been stuck on the rocks for three hours in great danger, and her keel was badly damaged. However, our friends were all safe, and very thankful we were for it.

An exhibition of dissolving views was given by one of the doctors in a large loft over the store at Battle Harbour, and it proved an immense success. The room was packed, people sitting on the floor, or standing wherever they could find room, for two whole hours. Their enjoyment can hardly be estimated by people accustomed to attend entertainments frequently. When it was over the audience expressed their willingness to stay for another two hours, a few even boldly declaring they'd be glad if it could go on all night! What would comfortable and *blasé* English playgoers have thought of this? People who depart with a jaded yawn at the descent of the curtain?

However, the next night a second exhibition of dissolving views was given, lasting three hours, and the two treats will doubtless furnish material for the whole winter's conversation.

One day we noticed a flag half-mast high on a neighbouring island. We knew the men from there were all away up the bay gathering wood for the winter, so we sent to offer aid. Alas! A poor woman had died in childbirth, and no help could now avail. It seemed to us very terrible when skilled and willing hands might have saved a valuable life. They had not sent before, they said, because they had always "done without a doctor" in such cases. The sufferings of the women through the ignorant treatment to which they are exposed surpasses anything we had hitherto imagined. But the people "don't hold with new ways" in that branch of nursing!

Our little wooden church often shook in the wind, for the gales are very strong, and might be alarming if we were not pretty used to storms. We all witnessed one day a most curious wedding taking place between an Eskimo lady and an Englishman. The bride was quite an old woman, attired in a dress of peacock blue, with a sash of a different colour tied in a bow with short ends on one side; two other women were similarly dressed. The man who gave the bride away was called the "father-giver," and he stated that he had given the lady to her first husband fifty years ago, and he meant next time to keep her for himself. The party also included one "bride's boy" and two "bride's girls," who were all married. The schoolmaster performed the ceremony, and made many comical mistakes. The bridegroom said "I will" before the proper time, and his promise, "with all my worldly goods I thee endow," was received with broad smiles, as he was known to own literally nothing! The bride, on the contrary, was a well-to-do woman, possessing a fish trap, salmon nets, half a schooner, and half a house. When the wedding party left the church several men fired off their guns, and followed to the quay firing all the way. A wedding where there is no firing is estimated as a very poor affair indeed.

### Nursing in Argentina.

TRAINED MALE AND FEMALE NURSES.—I.

The Argentine Republic, though not immaculate in all respects, has lately grasped the idea that trained nurses are desirable for the sick, and an Assistance Publique has been organised by the municipality of Buenos Ayres for the purpose of training male and female nurses for the sick. At present the course of training lasts but one year, and is confessedly incomplete, but as the organisers of the nurse-training school realise this, there is every reason to believe that it will be improved. It certainly seems to us that even of the short period of training too short a time is devoted to practical work. "Students must attend the hospitals for six months at least to acquire general practice; they may then devote themselves to the special line of diseases for which they wish to obtain a diploma." We certainly think special training should be based on more than six months previous knowledge, so much of that time must be spent, as Matrons know, in the mere "licking into shape" of the untrained, not to say raw, material provided. Also, it seems to us, that the minimum age—twenty—is fixed rather too low; but one must allow for the earlier maturity of the southern races, and as it is possible that the Argentine people have suffered from the tendance of elderly women who took up nursing because they were past earning their living in any other way, and incline, therefore, to err in the other direction. With regard to such women, however, we notice one very sensible arrangement. While they cannot be, and are not, interfered with, circulars are to be sent them, urging them to attend the lectures and obtain diplomas. The circulars in themselves may have little effect, but when the competition of the better trained nurse begins the untrained ones may bethink themselves of the opportunity of putting themselves on an equal footing. Moreover, we find among the temporary arrangements a "concert with the authorities to obtain that when once a certain number of those attending the school shall have received diplomas, others may be prevented from exercising their profession who have not followed the lectures and obtained diplomas."

In fact, the authorities of the nurse-training school of Buenos Ayres are determined to make trained nurses the rule, and not the exception, in the country. That they have an even wider aim may be inferred from the announcement that everyone who chooses may attend the lectures given between eight and nine p.m. on Saturdays on "First Aid in Cases of Accident." But it is to be hoped that they will take the necessary precautions to prevent those who have merely attended this course from posing as trained nurses, or grave discredit may be brought on the whole scheme. The lectures, however, seem to be of a thoroughly practical nature, if we may judge from the published syllabus, and anyone who attends them and makes the practical experiments given in the class will have the knowledge necessary to prevent much suffering before nurse or doctor comes. As in a mixed population like that of Argentina there will always be a demand for foreign nurses, it is arranged that persons holding foreign diplomas will be registered as belonging to the Assistance Publique on presenting a satisfactory certificate, or passing an examination to test their eligibility. It is intended when the general scheme has been put into satisfactory operation to grant special diplomas to midwives, masseuses, attendants on the insane, nurses for children's diseases, &c.

The remuneration offered is certainly good—three to four dollars a day for non-infectious and chronic diseases, or sixty dollars a month. No part of this is returned to the person employing the nurse, if for any reason the nurse's services are dispensed with, though if she (or he) is found not to suit another will be provided within twenty-four hours. The payment for acute, contagious, monthly, and mental cases is from four to five dollars a day. It is to be regretted that it is proposed, when the supply of nurses is found to be insufficient, to send out "the most experienced among the students" to attend the sick. In such cases it is expected that "the public will permit their attendance at the theoretical classes on Tuesdays and Saturdays from eight to nine p.m." Apart from the question of a student who has not had even a year's training being fit to go out as a responsible nurse, it may be doubted if the public, *i.e.*, the patient and his friends, will be ready to let the nurse go to lectures between eight and nine at night, we foresee endless friction from this arrangement. And, indeed, we do not see how a student, with the anxiety of an impending examination and the consequent importance of attending lectures on her mind, can attain the requisite concentration on the case in hand which belongs to the ideal nurse.

It must be admitted that the directors of the school seem to err in the direction of too great laxity. Thus, though it is set down in the rules that all nurses should be able to read, write, and keep accounts, we find in the third regulation for students that "those who cannot write may attend the classes as simple assistants until this deficiency shall have been remedied." Without putting too high an estimate on the "three R's," one may fear that these illiterates may go out with half-remembered scraps of information and bring disrepute upon the school. There is also an arrangement for students to be *externes*, not resident in the hospital, but going there for practice in the morning from six to eleven, which is likely to work badly. The discipline of the hospital is such an important part of training that we should not care to trust anyone who has not had it. Very few nurses will deny that the daily routine, the many little duties which in the eyes of the ignorant "have nothing to do with nursing," are in the result invaluable.

Nor do we think the course of study well arranged. Women begin their studies in the children's wards and those devoted to the diseases of women—the latter being surely among the places where the most skilled nursing is required. Male students begin in the lock and skin wards. These special courses finished, they go to the general medical and surgical wards, after which the women proceed to the maternity wards and the men to those for nervous diseases. A fortnight in an isolation hospital completes the training! It is fair to say, however, that the directors of the school do not consider this course of training as complete as could be desired; they hope in the near future to establish special courses of training for the nursing of children, the insane, and other special diseases. At present we cannot help feeling that the nurses turned out on such a system will leave much to be desired. But at the same time we recognise the importance of the initiative taken by the municipality of Buenos Ayres in founding a nurse-training school. Doubtless it will improve with time, as experience shows where weak points lie; and its very foundation will give an impetus to the nursing profession which we cannot doubt will in the end have good results.

### The R.B.N.A. Conversazione.

By One of the Visitors.

"I am dreadfully disappointed," exclaimed a clear voice, "If really am!" and the probationer looked ready to cry.

"Hush!" said her companion nervously, "somebody will hear you!" and she tried to look as if she herself did not share in the disappointment.

"I don't care if they do," rejoined the first speaker, "I I'd known that neither the Princess nor Mr. Corney Grain would be here I would never have come." Then luckily the music was heard beginning and the voice was drowned in the harmonies of the Bart.'s Musical Society, but not before a sympathetic smile had spread over several of the faces near.

The three rooms at Princes' Hall, which were devoted on the occasion in question to the service of the Royal British Nurses' Association contained, on Thursday, 7th inst., besides the usual crowd of ladies, a slight sprinkling of gentlemen, some of the former being evidently nurses wearing neat business-like costumes. A large proportion of the guests wore worldly garb, whilst a few ladies appeared in what appeared to be fancy nurse costumes, in all kinds of shades and shapes. For assuredly no earnest sick nurses would ever work in such fantastic caps as accompanied the many-hued gowns. There were a few matrons or sisters in uniform, and one or two besides who wore regular nursing caps with dresses which were in no sense of the word uniform.

On arrival the guests were received by Miss Stewart, Matron of St. Bartholomew's Hospital, attended by Mr. Pickering Pick and Sir Crichton Browne.

The refreshments were good and abundant, but the entertainment consisted only of the music, which was performed in the largest of the three rooms on the first floor. This was, therefore, inconveniently crowded, whilst the other two apartments were comparatively deserted. There were a great many Bart.'s nurses, and a few from various smaller hospitals, and those from Chelsea Infirmary and the Pay Hospital at Wandsworth were amongst the trimmest in costume. More private institutions were represented perhaps than hospitals, and certainly more provincial institutions than metropolitan ones. The gathering was said to be a smaller one than those of former years. Perhaps the knowledge that neither Princess Christian nor that popular entertainer, Mr. Corney Grain, would be present made some nurses, as well as their friends, think it hardly worth while to go to the expense of tickets. The objects of the Association were set forth on the programmes in company with an advertisement.

### Minor Appointments.

Drummond Union Workhouse Infirmary.—Miss Helen Taylor has been made Nurse of this infirmary. She was trained at Bradford Infirmary, and afterwards worked in Woodstock Union Infirmary, Oxford Poor-Law School, and as Staff Nurse at Wotton-under-Edge Nursing Institution.

Medical and Surgical Home, 39, York Place, Portman Square.—Miss Jeannie D. Garden has been appointed Sister-in-charge at this Home. She was trained at the Nightingale School, and was staff nurse and female theatre sister for nine years at St. Bartholomew's Hospital.

### Life in an Infectious Hospital.

(By a Nurse Who Worked There.)

In either general nursing or private life to understand fevers in their various forms and stages is most needful, and it is impossible to nurse a case of scarlet fever as it should be done without special training. In addition to giving good training in medical work, it is possible to learn a great deal about fevers in a comparatively short time, as the nurse's eyes, previously opened by experience, will see many things full of meaning for her, which would be lost on a perfectly untrained person. One reason why nurses do not complete their training by fever work, is from their holding an utterly false idea of what an infectious hospital is like. The management and organisation in most of them being now reformed, they are quite as comfortable, in fact, in some cases, more so than many general hospitals. Of course, the isolation is trying, and the disinfecting before going out is wearisome, but when a nurse has got into the swing of things she finds the life inside is one full of interest. Moreover, the grounds are generally large enough for a fair amount of exercise. It is more than three years since I spent seven months in one of the large London fever hospitals, after three years in a well-known provincial training school. Having an idea of going to the Colonies I thought a certificate for fever nursing would be of service, so in spite of many protests from my people, I accepted a post as charge nurse to a ward of 32 beds of scarlet fever patients, making up my mind that whatever the place proved to be like, I would work well for my certificate. I was pleasantly surprised on arrival. The wards were in separate blocks, connected by roofed open corridors. Between each ward and the next was a broad strip of grass, gravelled walks, and rows of sycamore trees. These made a very pleasant outlook for the wards, and were nice for the patients in the summer. The wards were long and broad, with plenty of windows, and a good space between the beds. The floors and furniture were scrubbed, and the chimneys were upright, which gave a rather cheerless impression to one accustomed to polished oak floors and Teale grates with underfloor chimneys, but I soon got accustomed to the change. The wards were worked by a charge nurse and two assistants, but an unfortunate system prevailed with regard to the latter. They were at any moment liable to be sent to fetch patients, sometimes being away several hours. This interfered very much with the work in the ward. Just as we had arranged the day's work, then the receiving-ward nurse would put her head in at the door and say, "ambulance, two minutes." Shortly after one nurse had gone the other might have to go too, and the poor charge nurse would be left with 30 or more patients, the dinner coming up, and the new cases to anticipate. These incidents were, however, rather exceptional, and can only happen in hospitals with ambulance stations.

There are extensive convalescent hospitals in connexion with fever hospitals, to which "robust convalescents" are drafted, so that experience of acute cases is the rule, and the long monotonous period when the patient is perfectly well, though isolated, is escaped by the workers in the hospital. The resident doctors are good in explaining things, and in insisting upon nurses learning how to describe the symptoms, the throats, &c., correctly, as well as the significance of complications. There are comparatively few doctors in proportion to the number of patients, so the nurses learn how to carry out the treatment for bad throats and to give vapour baths, &c. They also work in the diphtheria and enteric fever wards, as well as in those for scarlet fever. The nurses' quarters were well appointed, and the food excellent; in fact, everything was done for the comfort and happiness of the staff, and although life in a fever hospital may not be "a bed of roses," it is by no means the dreary, god-forsaken existence many people seem to imagine it! Even were it more fraught with danger and difficulty than it is, experience has proved to me that the game was well worth the candle.

### Causerie from New England.

### BOSTON, MASSACHUSETTS.

### An Epidemic.

Waltham, Massachusetts, having an epidemic of scarlet fever and diphtheria, was compelled to close for a time a majority of the public schools. The mortality was large, and the city was not provided with resources to meet the emergency. The City Council, forced to action by this state of affairs, recently appropriated 3,500 dols. to construct a pavilion for contagious diseases at the Waltham Hospital. Let us hope it will not verify the dictum of Dr. Thorn-Thorn, that wards constructed in times of panic are usually poorly and expensively built.

This hospital had a unique origin. The training school for nurses was established first, and the hospital followed—a method which reverses the usual plan. This hospital is a good example of what is known as "mill-construction," having the construction beams exposed, so as to prevent the creeping of fire between partitions. This plan is strongly advocated by the Hon. Edward Atkinson, a political economist of great practical wisdom.

### A New Hospital.

A new hospital, called the House of Providence, was opened last week in Worcester, the second city in Massachusetts. The municipality has its own general city hospital, and two of the larger State hospitals for the insane are located within its borders. It also has an excellent cottage hospital, called the Washburn Memorial Hospital. There are beds for fifty women and children in this admirably constructed building, which includes a circular ward. The House of Providence, as its name suggests, is a Roman Catholic institution in charge of the sisters. A large dwelling-house has been remodelled to accommodate twenty patients. Later, a more suitable hospital will be built with funds solicited by public appeals. A dispensary is now being added to the resources.

### Generous Bequests.

The widow of Dr. Ebenezer K. Hunt has bequeathed 25,000 dols. for the sole use of the Medical School of Yale University at New Havre, Connecticut. This school is small, and conducted with less vigour than many other parts of the University. As a memorial to her husband, she also provided for erecting in Hartford, Connecticut, a suitable building for the Hartford City Medical Society.

### Another New Hospital.

In October last, Norwich, a Connecticut city of twenty-three thousand inhabitants, opened a new hospital. It bears the not altogether euphonious name of the William W. Backus Hospital, in honour of a respected citizen who gave a considerable sum for the building, and who, by the terms of his will, made the hospital residuary legatee. The estate is now being settled, and the funds will soon be available. Another friend, Mr. William A. Slater, gave a still larger sum, but neither of the donors ever made known the extent of their benefactions. They simply let it be understood that they would give whatever amount was necessary to provide Norwich with a good hospital. Mr. Slater still lives to enjoy another of the fruits of his generosity—the Slater Memorial Hall, a building containing a fine audience-room, an art museum, art school, &c.

### Construction and Equipment.

The hospital is well located with plenty of air space and sun, and consists of six brick buildings, all of which are connected by basement corridors under cover, and some of them are also joined by open galleries on the outside. There is an administration building, four wards with ten beds each, in two separate buildings, an isolating ward with six beds and a few private rooms for paying patients, a nurses' home, and service building with kitchen, laundry, &c. Twelve beds are already occupied, and a small training school for nurses established. The women of Norwich became greatly interested in the hospital, and asked permission to supply all the linen. They notified the public of their readiness to receive contributions from any girl or woman, and the response came promptly from all sects, including Roman Catholics and Jews, and also from all social grades, from factory girls to women of leisure. The sums varied from five cents to 100 dols., and aggregated 1,700 dols. After buying and making the linen 500 dols. were left in the bank for further needs.

### Paying Patients.

The feeling that people do not value what costs them nothing, and the spirit fostered by our associated charities that giving people that which they are able to pay for tends to pauperism, led the Norwich Hospital authorities to establish the rule that patients shall pay something for treatment. Of course, the exceptional patients who are really very poor will be gladly received.

Another recent instance of the women of a city lending a hand in hospital improvements is found in Washua, New Hampshire, where, by a co-operative effort, they supplied the little hospital with a much-needed operating table.

### Electricity and the Ambulance Service.

A new adaptation of the electric street railway is an ambulance car. The springs are specially constructed to lessen the jar and shock as much as possible. Where the hospitals are situated on the line of the electric tramway this method of quick transportation might, perhaps, be advantageous. The experiment now being tried in St. Louis, Missouri, will be watched with interest. In view of the large number of accidents caused by the electric cars in crowded districts, the electric company might wisely include a few of these ambulances in their rolling stock. A practical hospital man thinks this plan might be a wise provision to meet some great calamity, but considers it unsuitable for regular use in conveying single patients.

### Everybody's Opinion.

[Correspondence on all subjects is invited, but we cannot in any way be responsible for the opinions expressed by our correspondents. No communications can be entertained if the name and address of the correspondent is not given, or unless one side of the paper only be written on.]

### NURSES AND ANÆSTHETICS.

"Enquirer" writes: Would someone kindly tell me if there be any special rule with regard to the administration of chloroform or other anæsthetics by a nurse? Is it allowable for a midwife to do it in the absence of the doctor? And is it considered right for a fully-trained and experienced nurse to administer the anæsthetic for a surgeon performing an operation? Having lately heard questioning on these points, I should be very grateful for a clear answer, and would like to see some correspondence on the subject.

*** The questions of our correspondent bear on points of such extreme importance to all nurses that we are glad to place before them the following answers, kindly furnished by one of the first authorities in London: (1) Certainly not. No nurse or other unqualified person should ever undertake the responsibility of giving an anæsthetic. (2) Under ordinary circumstances most certainly not. In remote country places and up-country in India, when a second qualified practitioner cannot be obtained to assist at an emergency operation, *e.g.*, for a strangulated hernia, the surgeon may depute a nurse or other unqualified person to give the anæsthetic; but his is the responsibility, and he so acts at his peril.

### Nursing in South Africa.

GRAHAMSTOWN.

Many changes have taken place recently in the Albany General Hospital here. The matron, Miss Magee, after a service of five years, has resigned on account of ill-health, and Miss Florence Burt, trained at St. Thomas's and at the Hospital for Epilepsy and Paralysis, succeeds her. A housekeeper—a new appointment—has been added to the staff, and a trained nurse from London is shortly expected. This hospital is rapidly coming to the front as a training-school for nurses, and other institutions will have to look to their laurels if they do not wish to be outstripped by this little institution. At the asylum a course of instruction has been carried on during the past year on the "Nursing and Management of the Insane." The medical superintendent is shortly to have the services of an assistant, which will leave him more leisure to devote to the theoretical instruction of the nursing staff. In the last annual report the withholding of certificates of competency to nurses until they have undergone a special training in lunacy is advocated. From the many letters which have appeared in the pages of The Hospital, it would seem that the nursing world out here is not overlooked, and neither is it, but women are strongly advised to hesitate before venturing to come out from England. South Africa may be said to be still in its infancy; it is a rough country, and unless a woman is strong, mentally as well as physically, and makes up her mind to "rough it," she had better stay at home. So often do we hear the complaint from nurses, "We were brought out here under false pretences," that it is well they should take timely warning once for all. The streets in South Africa are *not* paved with gold! All have to work, and work hard too, in a climate unsuited to muscular exertion, for a salary which at first glance looks liberal, but when the cost of living is considered is not much above what is obtained at home.

There is room for private nursing in all the larger towns, and in this city very frequently the doctors have to search in vain for a trained nurse, having often to be content with inexperienced girls or specimens of the "Mother Gamp" type, for of that race a fair number is still to be found even out here.

### Where to Go.

London Hospital, Whitechapel.—The Children's Christmas Tree Entertainment will take place on the afternoon of New Year's Day

Royal Institution.—On Thursday, December 28th, Dr. Dewar will give a lecture on Air.

The Dolls for the *Truth* competition, which every Christmas find their way into our London hospitals for the pleasure of the small patients, will this year, as last, be on exhibition at the Albert Hall on December 19th and 20th, where they will be well worth a visit of inspection.

### Presentation.

Miss Eliza Leek, Matron of the Bradford Eye and Ear Hospital, was presented with an illuminated address and a purse of £100 on her retirement from a post which she has filled for twenty-five years. The Chairman of the Hospital Committee spoke in high terms of the services which had been rendered by Miss Leek to the Institution, and many kind speeches were made by those assembled on this occasion.

### Notes and Queries.

**Queries.**

(228) *Hysteria.*—How can I get a patient suffering with hysteria into an institution?—*M. H. P.*

(229)—*Switzerland.*—Information wanted about nursing work in Switzerland.—*T. K.*

**Answers.**

(228) *Hysteria* (*M. H. P.*).—Consult the doctor as to the best place for the case.

(229) *Switzerland* (*T. K.*).—Write to Dr. Charles Kraft, whose papers have appeared in The Hospital.

### For Reading to the Sick.

NO MORE PAIN.

All the time life lasts we shall have to bear a greater or lesser degree of pain from the common ailments too trivial to mention, through the headache, the toothache, the rheumatic twinge, all of which spoil our pleasure, disturb our rest, affect our temper, and make our work burdensome, up to those attacks of fever which require medical attendance, put a stop to all business or pleasure, and leave us weak and low for weeks or months. Among the worst of these ills is a state of chronic ill-health, in which the poor sufferer seems neither to live nor die—that is, he does not progress either towards health or the grave. To such a one what comfort must the text give, "When the morning breathes there shall be no more pain." Yes, great comfort, if we do but believe in Christ and the joys promised us in another world; for it is only there, when our souls have crossed the narrow stream which separates us from the "undiscovered country," that there can be "no pain," and it is only there that all sorrow and sighing and tears will be wiped away. But how few of us can take the full consolation of the words? To many—nay, to most—death almost suggests annihilation, so vague an uncertainty have they as to what will follow when their souls have slipped from their earthly tabernacles. It will always be thus if we shrink from facing the inevitable. The wise man says in Ecclesiastes that one thing happeneth to all, high and low, rich and poor, the King and the beggar. It is quite right to seek to prolong life by every means in our power, to pray that God will take away our sickness and make us healthy and strong, but shall we be any more ready to depart in the future than we are now? This is a question we should ask ourselves. The truth is, that in our hope for the renewal of health and the removal of bodily disease, we are only driving off the evil day, without preparing ourselves to lay down our lives at Christ's call. We have abundant proofs in the Gospels, of our Lord's willingness to heal the sick—of His power to do so there is no doubt.

"At even when the sun was set,
The sick, O Lord, around Thee lay;
Oh, in what divers pains they met,
Oh, with what joy they went away."

They went away for the time perfectly well, but death still loomed in the future. At all times, then, we should prepare ourselves for it by humble prayer to the Almighty, and we cannot do better than use the words of Bishop Andrews, "We, remembering that the close of life may be near, beseech of Thee that Thou wouldst direct it in peace, sinless, shameless, and if it please Thee, painless, Lord, O Lord, gathering us together under the feet of Thine elect, when Thou wilt, and as Thou wilt, only without shame and sin." "When the morning breathes there shall be no more pain." In sickness or in death, our only refuge is Jesus the bearer of our sicknesses, the Almighty Healer of them.

### Novelties for Nurses.

KNITTED CAPES.

The Sanitary Knitted Corset Company, Mansfield Road, Nottingham, have prepared a most attractive catalogue of knitted wearing apparel, amongst which the knitted capes form a novel and useful feature. They should prove especially attractive to nurses, as they are a far better form of wrap than the shawl usually resorted to where open spaces have to be traversed, which frequently occurs in hospital life. The little capes are delightfully warm, and go into a very small space, the price being from 3s. 9d., according to size. In drawing especial attention to these capes, when the suggestion of a serviceable Christmas present is not unwelcome, we must not omit to draw attention to some other of the Nottingham specialities. The Combination bodice and petticoat has much to recommend it. It expedites dressing and does away with unnecessary layers of material and bands at the waist. Children's sleeping suits are most practically useful, as small limbs can remain warmly clad even when the offending bedclothes fall off. Knitted pantaloons for ladies are excellent for riding, or in place of extra petticoats, so inconvenient to the energetic. The catalogue contains so many ingenious contrivances that we advise our readers to glance at it for themselves.

### Trials of a Doctor.

By a Country Practitioner.

I am a doctor in a large, straggling, country village, with very few paying patients and a number of poor ones. Thus my time is well filled without adequate remuneration. I have often tried to devise some means of lightening my labours, but hitherto without success. A short time ago a brilliant idea seized me. I would organise a system of district nursing by means of the ladies of the neighbourhood. Within a week all the ladies in the parish had received notices of ambulance lectures to be held as soon as a class could be formed, and in another week the class had commenced. I found that I had to fill the post of lecturer myself, the subscriptions not being enough to pay anyone else. I consoled myself by reflecting, "It is only a little extra work for a short time;- eventually I shall secure extra leisure."

After two courses of lectures things seemed right for my project, so I convened a meeting of those who had obtained certificates, not quite so many as I had confidently hoped for, ten only of the class having done their teacher credit at the examinations, but these came at my call. I explained my wishes, which were taken up rapturously.

But now, when I was expecting rest and the end of my troubles, I found less rest and more trouble than ever.

Talk of ladies not being methodical and systematic! I can tell you there is enough system in the fair sex to drive a reasonable man crazy. The division and sub-division of labour among my ladies was something extraordinary. They elected among themselves a president, a vice-president, treasurer, secretary, head superintendent of materials, and working superintendent of materials. The number of committee meetings they had was terrific, and they expected me to attend them all. There was no use in my pleading important engagements; they promptly altered the hour of meeting to a time when I was free. Exasperated, at last I said I would come to no more, so they said, "If the doctor took no interest in the Nursing Guild it had better be abandoned at once." I felt inclined to agree with them, but as it would never do to have all the ladies in the village for enemies, I had to conciliate them. I said, of course, I would get up in the middle of the night to attend their committees if it would be any satisfaction to them.

At last they settled their uniforms, next to the committees the most important part of the whole affair, and I induced them to start work. I have drilled those ten nurses very thoroughly; I have inculcated perfect obedience. The first cannon is, "Send for the doctor"; the second is, "Do what the doctor tells you." It is well for my reputation and for my patients' health that this lesson has been thoroughly learnt, or else we should have a lively time with the coroner. The ladies will read medical books which they don't understand, from these they manufacture wonderful theories about the disease they happen to be nursing, and strive to bring their theories to bear upon their patients. They think they have discovered something of which the doctor is ignorant, and obedient to their training, they come instantly and tell him about it.

For instance, the other day Miss, or as she prefers to be called, "Sister" Jones, arrived in great excitement. "Doctor, do you know that Mrs. Evans' little girl has scarlet fever?" she asked. I replied, "I believe, Miss Jones, that she is suffering from bronchitis." "Indeed, Doctor, I am afraid you are mistaken; I have been reading about fevers, and I feel certain that little Susy Evans has the symptoms!"

I had a good deal of trouble too, with "Nurse" Fowler. She had unearthed from a second-hand bookstall a volume on medicinal plants, with recipes, and directions for their use, and she compounded whole bottles-full. She showed them to me one day with great pride. "I am going to take that to old Mrs. Kent to-day," she remarked, "my book says it is a sure cure for dyspepsia." "Yes," I answered, "the grave is a sure cure for most ailments. Please leave me to make up prescriptions for you, Mrs. Fowler, I can't afford to have my patients poisoned." She was dreadfully hurt, and didn't believe me a bit, but she threw away the precious decoctions, and with her husband's connivance the book fell into my hands and was burnt.

Another result of their indiscriminating reading is that my nurses find out they are suffering from every imaginable disease. The hero of Jerome's "Three Men in a Boat" suffered in the same way after spending a whole morning in company with a medical dictionary, but he arrogated to himself all the diseases he read of except the "housemaid's knee." My ladies are not so grasping; they find one at a time quite sufficient for them, but have it with all possible complications. But they are very easily cured. I have but to say to any one of them, "Dear me; now this is unfortunate. I had meant to ask you to kindly undertake a case for me." The fair patient rouses at once, and generally remarks she is feeling better. She thinks the fresh air would do her good, and it can't do her any harm to look in and see the sick person I have spoken of. Scarcely will the door close after me before the whilom invalid will put on her uniform, and spend the rest of the day attending to her "case."

Ah, well! my parish nurses are useful, and it is a comfort to think that my poor patients are well looked after, getting no end of dainty little dishes, what they call "nourishments" given to them. But, alas! for the leisure I anticipated. I have more work than ever before, and, worst of all, there are the committee meetings!

### The Guild of St. Luke.

(Communicated.)

Of all who live and labour,
We give the foremost place
To the best of all true workers,
The healers of our race.

Praise we the noble army,
The tender and the brave—
Who give their lives to rescue
Their fellows from the grave.

The selfless ones, the gentle,
The generous, the wise—
To these, the self-devoted,
We turn our grateful eyes.

When wintry blasts are sweeping
Across the snow-clad moor
They breast the storm to succour
The suffering, helpless poor.

In haunts of deadly fever,
In cellars, damp and lone,
They soothe the restless sufferer,
And still his anguished moan.

Their time, their rest, their leisure,
Their health and strength they give,
To heal the bruised and injured,
The suffering to relieve.

The calm and fearless surgeons,
Who live for friends and foes—
Show me the brave in battle
Who dare compare with those.

Not fired by fierce excitement,
By battle's blood-red glow,
By sight of falling comrades,
And face of angry foe.

But mid the after horror,
Beneath the dropping shot,
Calm head and hands must combat
The ill that war has wrought.

Sons of the Great Physician,
True followers of the Lord;
From Him, their Great Commander,
They win their sure reward.

### The Book World for Women and Nurses.

[We invite Correspondence, Criticism, Enquiries, and Notes on Books likely to interest Women and Nurses. Address, Editor, THE HOSPITAL (Nurses' Book World), 428, Strand, W.C.]

### CHRISTMAS BOOKS.

FIRST in order among the special books of the season come the bound volumes of magazines. Nearly everyone has an appetite for magazines, knowing by experience that it is rare to draw one quite blank, while in a year's supply the chance of finding entertainment is proportionately greater, and the serial story, if that is the attraction, can be read without the annoyance of a monthly break. Few publishers cater more agreeably for this taste than Messrs. Cassell and Co.

THE QUIVER for the current year is a sober, substantial volume, carrying a permit for Sunday reading into the severest household. Excellent and well-illustrated articles on topography and natural history supplement the theology for those who like something improving. And, for the weaker brethren, there is the attraction of stories, short and long, where lovers of varied degree play their parts with becoming moderation, and are sketched as they play it under the usual aspects. The illustrations to the shorter articles where the subjects are less fatally circumscribed, are full of grace and originality, and are sufficient in themselves to make the volume a source of entertainment.

LITTLE FOLKS hardly needs any commendation, so secure a position does it hold in the nursery where it is pre-eminently true that nothing succeeds like success. It may be hinted, however, that some of the hair-breadth escapes movingly depicted in the illustrations are of a nature to cause nightly tribulation to those sensitive little brains for whom wild beasts and Indians are ever-present dangers liable to lurk in every cupboard.

BO-PEEP, printed in clear large type, for quite the tiniest, is altogether without this blemish. It should prove the pleasantest of bridges from the griefs of learning to read to the joys of getting buried in a book for its own sake, and the most noisy member of the family would subside into a beatific calm if allowed a free hand with the paint box at the boldly drawn pictures on those wet mornings when the best regulated houses tend to become all nursery.

LITTLE MISS VIXEN is a readable little story of a naughty child, with a rather weak conclusion, tending to the theory that a passionate temper can be cured by a fortnight of discipline. It is hardly up to the author's usual mark.

A new edition of THE MASTER OF ST. BENEDICT'S seems to show that this novel has won a certain measure of success. Plenty of local colour, college slang, and indiscriminate flirtation blended together produce a romance which passes with the uninitiated for a picture of Cambridge life, and we venture to think that it is precisely this style of novel which is responsible for a good deal of the prejudice existing in quiet homes against a college career for women.

NICOLA, by M. Corbet Seymour, is a sketch of the career of a girl musician, written with a view to pointing out the emptiness of even realised ambition and the value of home ties and affection, however humble. It is a quiet, unpretentious story with a healthy tone, and is very prettily bound and illustrated.

BOOKS NOTICED.—"The Quiver, 1893" (Cassell and Co.), "Little Folks, 1893" (Cassell and Co.); "Bo-Peep" (Cassell and Co.); "Little Miss Vixen," by E. E. Green (Oliphant,

**THE BOOK WORLD FOR WOMEN AND NURSES**—*continued.*

Anderson, and Ferrier); Nicola," by M. Corbet Seymour (Blackie and Son); "The Master of St. Benedict's," by Alan St. Aubyn (Chatto and Windus).

### CHRISTMAS NUMBERS.

There can be small doubt when the magazines are compared this Christmas that the Americans are still far ahead in the art of illustration. The Century is a remarkably good number. The reproductions of Rembrandt alone will repay long study. But indeed every illustration tells us a story in itself, and is distinguished by some special note of interest or technical skill. "Christmas at the Children's Hospital," a full-page illustration by J. Carrell Lucas, is the outcome of a competition instituted by The Century for art students with a view to encouraging the art of illustration, and is a very interesting composition. The artist has perhaps not quite succeeded in banishing the inevitable monotony of a glimpse of beds and bare walls, but he has vanquished the temptation to lend his patient a fictitious air of well-being, and has found evidently a real sick boy for his model. There are drawn lines of pain across the brow, and the teeth are just showing in the uneasy attempt at a smile as the smart lady visitor shows off her toys, while the eyes have the strained, unhappy look which nurses know so well as an indication of actual suffering. The inevitable orange and bottle of physic, which are part of the stock in trade of the sick-bed artist, are not wanting, nor an æsthetic nurse with high sleeves and a dainty cap.

The best thing in Harper's Magazine is "The Two Gentlemen of Verona," charmingly illustrated by Mr. Edwin A. Abbey, with a comment, garrulous, indistinct, but entertaining, by Mr. Lang. There are some good nigger stories for those who are not disheartened by phonetic spelling, and some pretty sketches of life in the Old Dominion. The smaller illustrations to the story of Colonel Blood's career, by Mr. Howard Pile, are good examples of fine effects rendered in a few strokes of the pen.

Scribner's Magazine has more than its lawful share of interest this Christmas in having secured an unpublished work of Scott's. It consists of a series of private letters supposed to have been written in the time of James I., and continued for his own amusement by Scott until Lockhart and Ballantyne persuaded him to use the subject matter for a romance, and he turned his mind to the "Fortunes of Nigel" instead. The letters were intended as a jest to deceive "a few musty antiquaries," but they have a very substantial interest of their own apart from the occasional suggestions to be encountered of Nigel and his fellows. Some beautiful drawings of Della Robbia Monuments from various towns in Italy form the principal attraction in the way of illustrations to this number.

The English Illustrated has taken on quite a new appearance under its fresh management. Rather a pleasant effect is produced by the blue and red tinting of some of the illustrations. The glimpses of Venice are very successful, and the reproductions of photographs of ancient earthworks at Casterbridge are good, with the rare quality of fully elucidating the text. As regards the rest of the number, it must be candidly said that quantity rather than quality is the leading feature.

### Sylvia's Home Journal.

Sylvia's Journal contains several interesting tales and articles. "The Moon Slave," by Barry Pain, is a quaint and prettily told fairy tale. This commences the number. Mrs. Alfred Hunt's "Grass of Parnassus" is a pleasing story of a young lady who knew her own mind in spite of appearances being against her. The illustrations are very well produced, and the fashions and fancies have also their place in these pages.

